# Conflicting Dynamics of Galling and Pollination: *Arastichus gallicola* (Hymenoptera, Eulophidae), a Specialized Parasitic Galler in Pistillate Flowers of *Thaumatophyllum bipinnatifidum* (Araceae)

**DOI:** 10.3390/plants13243520

**Published:** 2024-12-17

**Authors:** Sergio Jansen-González, Simone P. Teixeira, Rodrigo A. S. Pereira

**Affiliations:** 1 Programa de Pós-Graduação em Entomologia, Faculdade de Filosofia, Ciências e Letras de Ribeirão Preto, Universidade de São Paulo, Ribeirão Preto 14040-130, SP, Brazil; 2 Escuela de Biología, Centro de Investigación en Biodiversidad y Ecología Tropical, Universidad de Costa Rica, San Pedro de Montes de Oca, San José 11501-2060, Costa Rica; 3 Departamento de Ciências Farmacêuticas, Faculdade de Ciências Farmacêuticas de Ribeirão Preto, Universidade de São Paulo, Ribeirão Preto 14040-903, SP, Brazil; spadua@fcfrp.usp.br; 4 Departamento de Biologia, Faculdade de Filosofia, Ciências e Letras de Ribeirão Preto, Universidade de São Paulo, Ribeirão Preto 14040-130, SP, Brazil; raspereira@usp.br

**Keywords:** Chalcidoidea, gall-inducing insects, gall development, insect–plant interactions

## Abstract

In the complex dynamics of plant–insect interactions, the specialized galling of reproductive structures presents unique evolutionary adaptations. This study investigates the parasitic relationship between *Arastichus gallicola* (Hymenoptera, Eulophidae), an ovary-galling wasp, and the inflorescences of *Thaumatophyllum bipinnatifidum* (Araceae). We employed field experiments and histological analyses to investigate the mechanisms driving this interaction. We reveal that ovule fertilization is not required for gall formation; however, pollination substantially enhances gall retention by reducing inflorescence abscission. Inflorescences exposed solely to galling presented a 64% abscission rate, whereas those with both galling and pollination experienced 33% abscission, underscoring pollination’s role in mitigating inflorescence loss. Detailed observations of *A. gallicola* oviposition and larval development reveal the intricate gall formation process characterized by progressive tissue hypertrophy surrounding the larva. Galling and seed development were mutually exclusive, with only 9% of fruits containing both. This mutual exclusivity suggests a competitive interaction for developmental resources within the ovary. Our findings underscore the specialized larval biology of galling chalcid wasps, illustrating how interactions between gall formation and host reproductive strategies shape the evolution of gall induction in floral tissues. Our study advances the understanding of ovary-galling adaptations and the selective pressures shaping antagonistic and mutualistic interactions in plant reproductive structures.

## 1. Introduction

The interaction between plants and gall-inducing insects represents one of the most intricate and intimate plant–animal relationships. Gall formation, typically induced by holometabolous insects, begins with the female insect ovipositing into specific plant tissues, followed by the development of the larva within those tissues [1,2]. This process’s success mainly depends on the female’s ability to select the appropriate target tissue and initiate gall formation, which protects the egg and the first larval instar. This initiation is facilitated by tissue damage during oviposition and the injection of hormone-like substances or elicitors [3,4], potentially involving molecular or microbiological factors [5]. The selected tissue is usually in a physiologically reactive, undifferentiated stage, making it particularly susceptible to the gall-inducing factors introduced by the female and the subsequent larval secretions [6]. These factors induce cell division, tissue de-differentiation, hypertrophy, and the localized accumulation of nutrients and chemicals around the developing larva [7]. This intricate manipulation ensures that the gall remains attached to the host plant, promoting its continued growth, maintenance, and modification in favor of the galler.

In this study, we assessed the interaction between *Arastichus gallicola* (Zhang, Gates, Hanson & Jansen-González, 2022), a chalcid wasp (Eulophidae), and the inflorescences of *Thaumatophyllum bipinnatifidum* (Schott ex Endl.) Sakur. and Calazans & Mayo (Araceae). These species were selected as they represent a suitable system for investigating the relationships among pollination, inflorescence-abscission risk, and gall formation. The wasp was initially identified as belonging to the genus *Exurus*. However, a recent study reclassified it as a new genus, *Arastichus*, associated with the inflorescences of *T. solimoesense* (A.C. Smith) Sakur., Calazans & Mayo, and *T. bipinnatifidum* [8,9].

The difficulty of gall induction varies depending on the location and origin of the target tissue. Vegetative structures, which are generally less complex at the cellular and ontogenetic levels, are likely less challenging to gall than reproductive structures, where tissues of both gametophytic and sporophytic origins coexist within a single organ, such as flower ovaries or seeds [10,11,12]. The structural complexity of reproductive tissues not only complicates gall formation but also increases the risk of abscission, particularly when the retention of the organ hosting the gall depends on external processes like pollination, fertilization, and embryogenesis [10,13,14]. Consequently, it is unsurprising that surveys frequently report more insect galls in vegetative tissues than in reproductive tissues [15], a trend observed even in so-called “super host” plants [16]. These findings support the notion that galling reproductive structures is inherently more challenging, requiring additional adaptations to deal with complex ontological processes to mitigate abscission risk.

The mega-diverse superfamily Chalcidoidea (Hymenoptera) represents one of the few insect groups that have successfully evolved strategies to gall plant reproductive tissues. Despite most chalcid species being entomophagous parasitoids, gall-inducing habits have independently arisen in this group [17]. Phytophagous chalcid gallers exhibit a fascinating spectrum of interactions, ranging from highly refined mutualism to parasitism, each with distinct strategies to deal with the complexities of tissue ontogeny and the risk of abscission. The interaction between fig trees (genus *Ficus*) and fig wasps (Hymenoptera, Agaonidae) suggests that pollination and larval development strategies play a role in mitigating inflorescence abscission. In this obligatory nursery pollination mutualism, the wasps induce galls within ovules with just-fertilized embryo sacs, synchronizing their larval development with the early stages of plant embryogenesis. Inflorescence abscission is prevented by the pollination facilitated by the agaonid wasps, which specialize in galling the nucellus and endosperm. Thus, the wasp larvae rely strongly on the plant’s embryological processes to complete their development [10].

Among fig wasps, certain non-pollinating fig wasps (NPFWs) from the families Epichrysomallidae and Pteromalidae also induce galls in fig ovaries. In NPFWs, synchronization between larval development and plant embryogenesis occurs; however, gall induction occurs in the nucellus, an ovular tissue whose formation does not depend on fertilization. This allows these wasps to adapt their development to ovule conditions irrespective of whether fertilization occurred [11,18,19]. The risk of abscission, in this case, is reduced either by ovipositing in pollinated fig flowers or, in the absence of pollination, through an as-yet-unknown mechanism where a higher number of galls appears to correlate with a lower likelihood of abscission [9]. Conversely, for chalcid seed predators, the strategy involves ovipositing in fruits and seeds at their early development stages. The larva then grows alongside the developing seed without notably disrupting embryogenesis and consumes the seed at a more advanced stage, when the risk of abscission is reduced [20,21]. The distinction between seed predation and gall-inducing feeding strategies is often blurred, as some species’ larvae can induce hypertrophy in seed tissues, resembling the effects typically caused by true gallers [21].

The described adaptive strategies suggest no universal mechanisms govern inflorescence-abscission mitigation across different plant–insect systems. Further investigation into additional insect–plant interactions is necessary to identify the mechanisms shaping the dynamics of gall induction and inflorescence-abscission prevention. Therefore, we employed field experiments and developmental studies involving flower dissection and histological analysis to investigate the galling process and the relationship between pollination, inflorescence-abscission risk, and gall formation. We demonstrate that *A. gallicola* is a specialized ovary galler in *T. bipinnatifidum* plants. We also reveal that ovule fertilization is not required for gall formation, with pollination playing a crucial role only in preventing inflorescence abscission.

## 2. Results

### 2.1. Effects of Pollination on Inflorescence Retention

The abscission frequency significantly differed across the experimental treatments (χ^2^ = 27.87, degrees of freedom = 3, *p* < 10^−3^). The treatment in which flowers were neither pollinated nor exposed to female wasps resulted in 100% inflorescence abscission. Flower pollination resulted in a 33% inflorescence-abscission rate, regardless of wasp exposure. In the treatment where flowers were exposed to wasps but not pollinated, the abscission rate was 64% (Table 1).

The mean number of fruits produced per inflorescence did not differ significantly across treatments, ranging from 231.2 to 313.5. In the treatments where flowers were pollinated, the number of fruits containing seeds was, on average, 33% higher in the inflorescences not exposed to the wasps. Regarding the wasp-exposed treatments, unpollinated inflorescences produced 40% more fruits exclusively containing galls. Few fruits (10.3%) produced a combination of galls and seeds in the treatment where flowers were pollinated and exposed to the wasps. The number of empty flowers, i.e., those containing neither seeds nor galls, did not differ significantly across treatments, ranging from 25 to 30 on average (Table 2).

### 2.2. Female Oviposition, Larval Development, and Gall Formation

**Figure 1:**
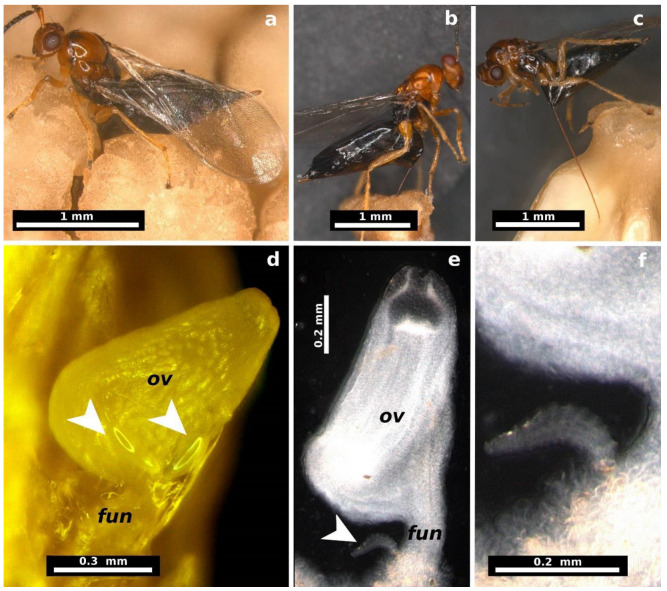
Oviposition in *Thaumatophyllum bipinnatifidum* flowers and early developmental stages of *Arastichus gallicola*. (**a**) Close-up of female wasp during oviposition; (**b**,**c**) ovipositor insertion sites: (**b**) through the flower stigma and (**c**) through the ovary wall; (**d**) detail of egg placement, with arrows indicating two eggs attached to the ovule base, each anchored to the funicle by a long peduncle; (**e**) position of the first larval instar (arrow) near the ovule funicle; (**f**) close-up of the first larval instar. Abbreviations: *fun* = ovule funicle, *ov* = plant ovule.

**Figure 2:**
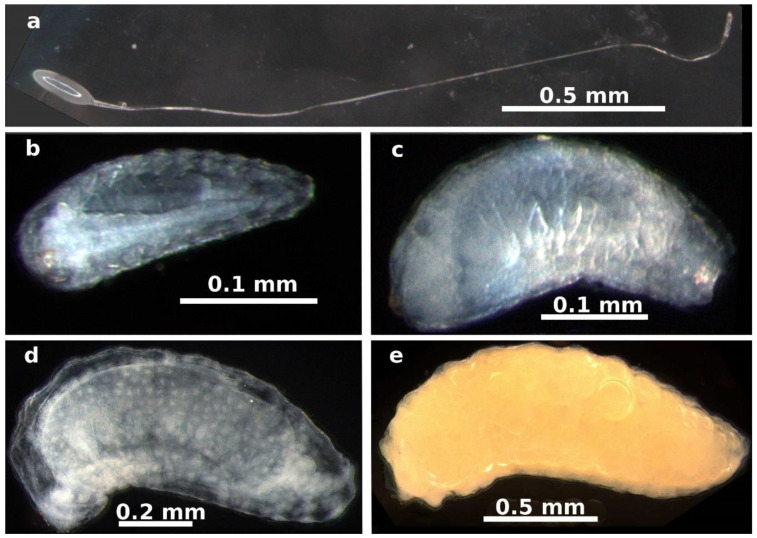
Immature stages of *Arastichus gallicola*. (**a**) Egg with peduncle; (**b**) first larval instar at 7 days; (**c**) first larval instar at 17 days; (**d**) second larval instar at 25 days; (**e**) third larval instar at 37 days.

**Figure 3:**
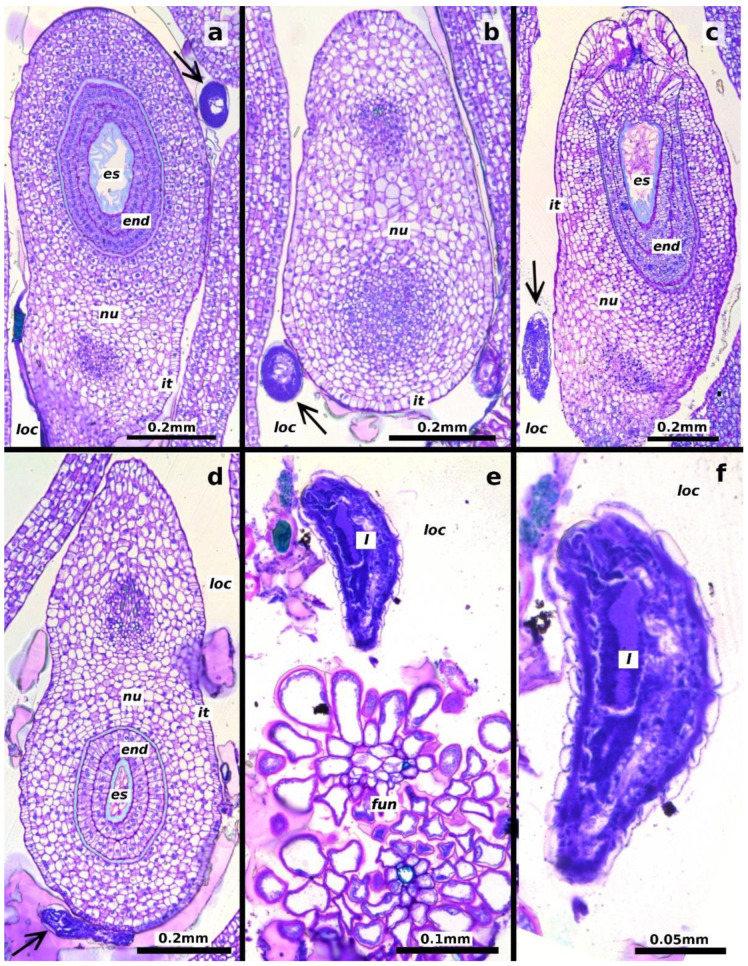
Longitudinal sections of *Thaumatophyllum bipinnatifidum* ovules showing early developmental stages of *Arastichus gallicola*. (**a**,**b**) Position of the wasp egg (arrows) relative to the ovule; (**c**–**f**) position of the first-instar larva (arrow) relative to the ovule; (**e**) detail of the larva near elements of the ovule funicle; (**f**) close-up of the larva. Abbreviations: *end* = endothelium, *es* = embryo sac, *fun* = funicle, *it* = inner integument, *l* = larva, *loc* = ovarian locule, *nu* = nucellus.

Adult females inserted their ovipositors through the stigma or ovarian walls of the pistillate flowers (Figure 1a–c). The egg stage was observed from 1 to 5 days after oviposition. The eggs are elliptical (length: 0.218 ± 0.022 mm, width: 0.070 ± 0.007 mm, mean ± SD, *n* = 20 eggs) with a long pedicel several times longer than the egg (Figure 2a). Eggs are deposited individually or in groups of up to four within the locular space, each attached to the ovule funicle by its long pedicel (Figure 1d and Figure 3a,b).

We identified three larval instars by observing molts at two stages of larval development, where the cephalic capsule of the previous instar remained attached to the larva’s body.

The first instar began five days after oviposition. At this stage, the larva was attached to the ovule funicle within the ovarian locule, likely feeding on the mucilaginous material filling the locule (Figure 1e,f and Figure 3c–f). The larvae at this stage measured 0.312 ± 0.097 mm in length and 0.113 ± 0.050 mm in width (mean ± SD, *n* = 60 larvae, Figure 2b).

**Figure 4:**
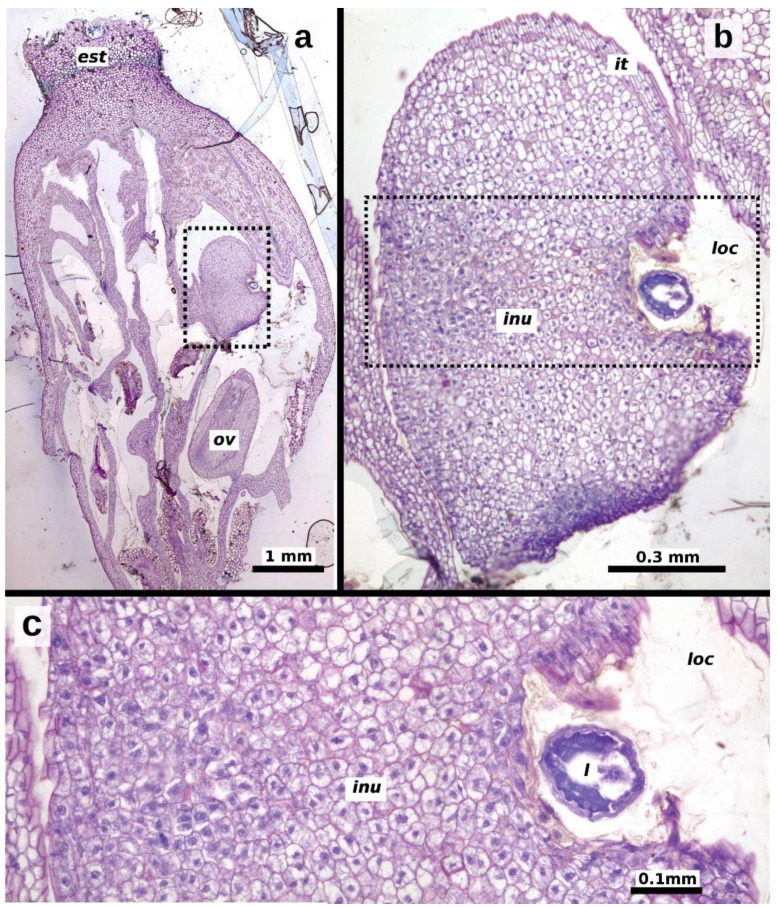
Longitudinal sections of *Thaumatophyllum bipinnatifidum* ovaries, showing an ovule undergoing gall formation and an unaffected ovule (ov), 20–25 days after oviposition. (**a**) Pistillate flower with affected (dotted rectangle) and unaffected ovules; (**b**) close-up of the affected ovule, showing the ovule elements; (**c**) close-up of the dotted area in (**b**), highlighting hypertrophied cells surrounding the larva, indicative of gall formation. Abbreviations: *inu* = induced nucellus, *it* = inner integument, *loc* = ovarian locule, *ov* = unaffected ovule.

**Figure 5:**
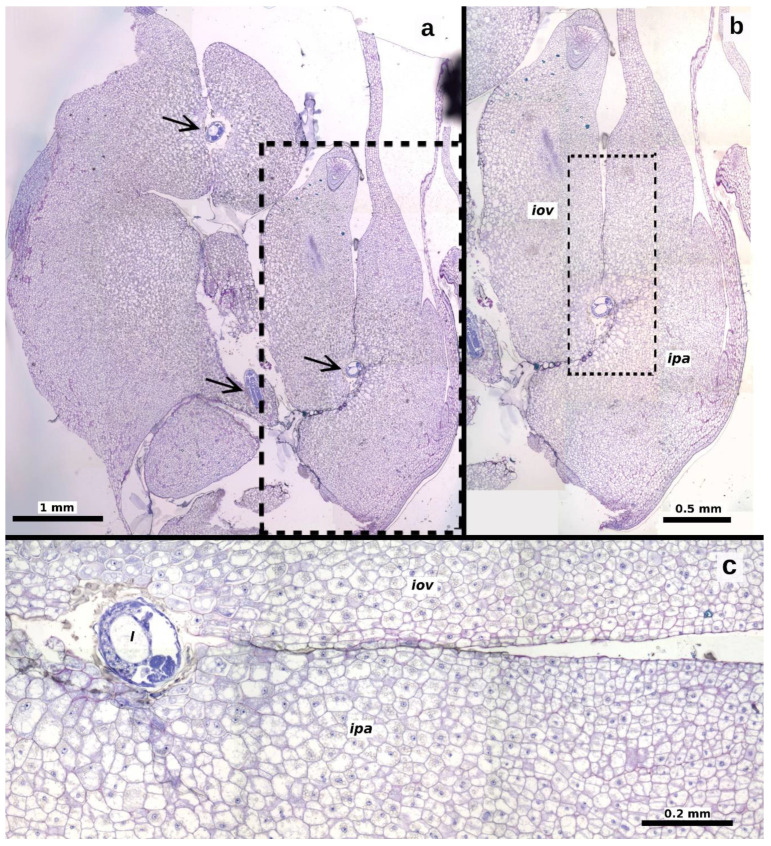
Longitudinal sections of a *Thaumatophyllum bipinnatifidum* ovary with galled ovules containing larvae (arrows). (**a**) Overview showing three larvae within ovule galls; (**b**) close-up of the dotted area in (**a**), detailing the affected ovule and parenchyma; (**c**) close-up of the dotted area in (**b**), illustrating hypertrophied cells throughout the tissue surrounding the larva. Abbreviations: *iov* = induced ovary tissue, *ipa* = induced parenchymal elements, *l* = larva.

The second instar was observed starting 20 days after oviposition, with larvae measuring 1.193 ± 0.471 mm in length and 0.475 ± 0.172 mm in width (*n* = 40 larvae, Figure 2c). The gall began to form at this stage, characterized by hypertrophy of the plant ovule tissues to which the larva was attached (Figure 4). The increase in ovule volume was driven by disorganized cell division within the ovule’s integuments and nucellus. Similar modifications were observed in the cells of the locular septa and the ovary walls adjacent to the larva (Figure 5a,b). These cells exhibited enlarged vacuoles and starch grains in the cytoplasm (Figure 4b,c). As the modified tissue grew, it enveloped the larva, forming gall tissue composed of hypertrophied cells from the flower ovule and adjacent tissues (Figure 5c).

**Figure 6:**
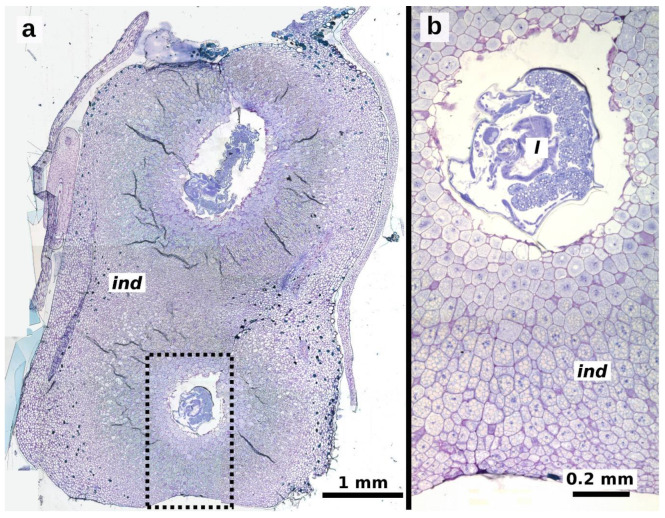
Longitudinal sections of fully developed galls of *Arastichus gallicola* in *Thaumatophyllum bipinnatifidum* ovary. (**a**) Section showing a galled ovary with two galls, each containing a larva; (**b**) close-up of the dotted area in (**a**), highlighting induced parenchymal elements. Abbreviations: *ind* = induced parenchymal elements, *l* = larva.

The third instar was observed 30 days after oviposition, with larvae measuring 2.407 ± 0.562 mm in length and 0.791 ± 0.137 mm in width (*n* = 40 larvae, Figure 2d,e). By this stage, the gall was fully formed, and the larva continued to feed on the hypertrophied tissue within the gall (Figure 6a,b). Pupation occurs inside the gall, and adult wasps emerge when the spathe detaches from the ripe infructescence.

## 3. Discussion

Our study demonstrates that *Arastichus gallicola* is an ovary galler in the aroid *T. bipinnatifidum*. Insect eggs can be deposited in multiple ovules, with a single flower ovary capable of bearing several galls. This finding is consistent with previous observations that *A. gallicola* can induce multiple galls within a single ovary of *T. solimoesense* [9]. The females of *A. gallicola* oviposit within the ovaries of the pistillate flowers during anthesis, with the eggs being precisely attached to the ovule funicle. The precise egg deposition likely plays a crucial role for chalcid wasps specialized in galling plant ovarian structures. Similar sophisticated oviposition strategies have independently evolved in three chalcid families associated with fig trees, including the pollinating fig wasps (Agaonidae), the *Idarnes* species group *flavicollis* (Pteromalidae), and the genus *Sycobia* (Epichrysomallidae) [19,22]. In these lineages, regardless of whether the wasps oviposit from within the fig cavity or from the external fig surface, the ovipositor is inserted through the flower style, and the egg is deposited near the stylar canal entry [10,11]. These observations support the hypothesis that precise ovipositor insertion is essential to ensure undisturbed and successful larval development.

The larva of *A. gallicola* appears to be responsible for the gall-inducing process, as the formation of undifferentiated and hypertrophied gall tissue with a parenchymatic appearance begins when the larva changes to the second instar and comes into contact with the ovule and surrounding ovarian tissues, such as the septa and walls. Gall-inducing insects secrete effector molecules, including phytohormones, which stimulate surrounding tissue growth [3,4,23,24]. Borges [25] highlights that these insects produce auxins and cytokinins at levels manifold higher than those in ungalled plants, creating localized hotspots of tissue growth. A recent study suggests that gall induction by a cecidomyid insect in cassava involves the integration of bacterial DNA vectorized by the insect into the plant’s genome. The bacterial DNA was linked to genes influencing plant cell transformation and the ubiquitin–proteasome system, a regulatory mechanism involved in protein turnover and developmental processes [5]. In the case of *A. gallicola*, gall formation begins approximately 20 days after oviposition. The delay between oviposition and gall formation suggests that the mechanical stimulus from ovipositor insertion or any substances injected by the female during oviposition plays a limited role in initiating the gall. However, Elias et al. [26] demonstrated that venom glands in gall-inducing and non-galling fig wasp species have distinct peptide profiles, suggesting that the secretions injected during oviposition by gall-inducing species may play a role in modulating plant tissue and initiating gall formation.

Although the gall-inducing habit has evolved multiple times in six Chalcidoidea families [17], to the best of our knowledge, ovary chalcid gallers have been confirmed only in wasp species associated with fig trees and the aroid genus *Thaumatophyllum*, although ovule gall induced by the Ichneumonoidea *Allorhogas uberlandiensis* Joele & Zaldívar-Riverón, 2019 has been reported for *Miconia chamissois* Naudin (Melastomataceae) [12,27]. Additionally, seed predation without inducing abnormal tissue growth is known in genera such as *Megastigmus* (Torymidae: Megastigminae) and *Bephratelloides* (Eurytomidae) [20,21]. In the pollinating fig wasps and seed predators, which depend on embryo sac fertilization (in angiosperms) or megagametophyte development (in gymnosperms), the larvae employ a koinobiont strategy, allowing the seed to continue growing while feeding on its nutrient-rich tissues. This strategy likely evolved to avoid disrupting the proper embryonic development of the plant. In these instances, during the initial larval development, pronounced cellular modifications in the ovarian tissues in contact with the larva are not observed [10,28]. Conversely, in galling species that do not require pollination for larval development, such as *Idarnes* [11] and *Arastichus* (present study), the modification of the ovarian tissues surrounding the larvae is pronounced, resulting in aggressive gall development characterized by rapid and disorganized cell division.

Lack of pollination often leads to the abscission of flowers or inflorescences [14]. The risk of organ abscission may represent a substantial selective pressure on gallers associated with the reproductive structures of plants. This assertion is supported by the evidence that the capacity to avoid abscission has evolved in all chalcid galling lineages that do not depend on fertilized embryo sacs for larval development. Our experimental study confirmed that inflorescences with unpollinated flowers of *T. bipinnatifidum* abscise readily. However, our findings also demonstrate that unpollinated inflorescences containing galls of *A. gallicola* are less likely to abscise than solely unpollinated inflorescences, likely due to an unknown sink effect directed toward the galled organ [29].

A similar effect on preventing abscission is observed in Chalcidoidea and Ichneumonoidea wasps. Several *Megastigmus* species associated with Pinaceae species lay eggs inside the developing ovules of their host conifers before pollination occurs [20]. In *M. spermotrophus*, it has been demonstrated that the megagametophyte containing the insect larva continues to develop even without pollination [28]. Introduced *Ficus* species also provide evidence that ovary gallers can prevent inflorescence abscission. For instance, in Brazil, *F. benjamina* and *F. microcarpa* were introduced for ornamental purposes, and their associated wasps arrived decades after these introductions. The first species to recolonize these plants were ovary-galling NPFWs (*Sycobia hodites* and *Walkerella microcarpae* in *F. benjamina* and *F. macrocarpa*, respectively), which could prevent the abscission of unpollinated figs [18,30,31,32]. The ability to prevent fig abscission has also been experimentally demonstrated in a species of the *Idarnes* group *flavicollis* associated with the neotropical *F. citrifolia* [11,33]. Similarly, *Allorhogas* sp. (Ichneumonoidea), which induces galls in *Miconia calvescens* (Melastomataceae), also prevents fruit abscission, as higher rates of infestation were observed in plants with advanced phenology, where most fruits were mature [34].

We demonstrated that pollination influences the interaction between *T. bipinnatifidum* and *A. gallicola*. Although *A. gallicola* can induce galls in ovules with unfertilized embryo sacs, pollination enhances the insect’s reproductive success by significantly reducing the risk of inflorescence abscission. However, when wasps were exposed to pollinated flowers, they succeeded in inducing galls in approximately half of the available flowers. Moreover, gall formation and seed development were mutually exclusive events, with only 9% of the fruits containing both galls and seeds. While our study design did not allow us to uncover the mechanism behind this pattern, it suggests that seed formation may interrupt gall development, or conversely, gall formation may inhibit seed development. Notably, the rate of fruit abortion (i.e., fruits not producing seeds or bearing galls) did not significantly differ across the experimental treatments, suggesting that the experimental conditions did not lead to differential larval mortality or seed abortion.

Our findings on the negative relationship between seed development and gall formation provide valuable insights into the selective pressures that may drive the evolution of the ovary-galling habit in insects and offer a plausible hypothesis for why this life history strategy has emerged in a limited number of biological systems. Within the framework of embryo sac fertilization negatively impacting gall development and vice versa, insects are expected to be individually selected to oviposit as early as possible in the flower ovaries before pollination occurs. Conversely, plants might be selected to limit exposure of their flower ovaries by investing in mechanical barriers, such as the aroid spathe, which restricts access to a brief window during anthesis, or by synchronizing flowering phenology at the population level to constrain the maintenance of insect populations year-round. While physical barriers may not universally prevent ovary-galling, as evidenced by the sophisticated oviposition behavior of chalcid wasps, facilitated by the complex structure of their ovipositors [35,36], phenological adjustments appear to be more effective. Notably, ovary-galling is observed predominantly in plant groups associated with host-specific pollinators and a year-round flowering pattern, such as *T. bipinnatifidum* (present study) and fig trees [37].

Under natural conditions, however, the prevalence of *A. gallicola* is likely limited by other insects interacting with *T. bipinnatifidum*. In first place, there is a physical interference with the pollinating dynastid beetles, *Erioscelis emarginata* (Mannerheim, 1828) [38], which gather around the pistillate flowers in large numbers during the same period that *A. gallicola* oviposits and disrupt and limit the wasps’ ability to oviposit. The parasitoid *Prodecatoma philodendri* Ferrière, 1924 (Eurytomidae) can also exert control over *A. gallicola* populations [8,9]. Furthermore, closing the spathe and the subsequent filling of the cavity between the spathe and the spadix with fluid further restricts the oviposition opportunities for any remaining female wasps inside the inflorescence.

Our study advances the understanding of ovary-galling insects by highlighting the selective pressures on both plants and insects, which can lead to divergent evolutionary outcomes, such as mutualistic brood site pollination or antagonistic seed predation. The dependence on pollination seems to play an essential role in determining these pathways. For instance, it has been speculated that the agaonid *Ficus* pollinators evolved from a gall-inducing chalcid ancestor [39]. The urn-shaped inflorescence in the Moraceae clade formed by *Ficus* and Castilleae [40] likely drove the evolution of insect biology. This particular inflorescence shape may influence how floral resources are exploited and, in turn, shape the traits of the associated insects. If pollination enhances the larval success of ancestral gallers, pollination behavior could evolve as a beneficial trait. Conversely, in scenarios where pollination does not directly impact larval success, as in plant groups previously associated with specialized pollinators such as scarab beetles and aroid plants [41], the galler interaction may evolve into an antagonistic relationship. Our findings highlight the importance of considering the intricate ecological and evolutionary contexts when examining the relationships between ovary-galling insects and their host plants.

## 4. Materials and Methods

### 4.1. Study Site and Species

The experiments were conducted at the University of São Paulo campus in Ribeirão Preto (21°10′ S; 47°48′ W), Brazil, between 2009 and 2011. The campus features extensive gardens and lawns of various spontaneous and cultivated plant species. The local climate falls under the Aw category (Tropical Savanna) according to the Köppen classification, characterized by wet summers and dry winters. The lowest average monthly temperature is recorded in July (19.5 °C), while the highest occurs in October (24.8 °C). Annual rainfall averages 1384 mm, with January being the wettest month (mean of 256 mm) and July the driest (mean of 21 mm) [42]. The field study was conducted during the rainy months (i.e., September to March)

*Thaumatophyllum bipinnatifidum* plants grow naturally as scandents or hemiepiphytes, blooming year-round with individuals consistently producing inflorescences. The flowers are arranged on a condensed, finger-like spadix, surrounded by a spathe (bract). The pistillate flowers are in the lower portion of the spadix, while the staminate flowers are positioned in the upper portion. The spathe remains closed until anthesis, during which a strong, sweet scent is released, attracting both its beetle pollinators and female *A. gallicola* wasps [38,43]. The wasps arrive earlier than the beetles, entering through the first available opening, and reach the base of the inflorescence where the pistillate flowers are located to oviposit in them. The spathe closes approximately 24 h after anthesis, forcing the pollinators out as the staminate flowers release pollen. About 36 h after anthesis, the spathe closes completely, and the cavity between the spathe and the spadix fills with a fluid secreted by the plant. Months later, the spathe develops a dehiscence line at its base and detaches, revealing the ripe infructescence [8,9].

### 4.2. Effects of Pollination and Wasp Oviposition on Inflorescence/Infructescence Retention

To evaluate the effects of pollination and wasp oviposition on inflorescence and infructescence retention and fruit/gall development, we conducted experiments using controlled combinations of pollination and oviposition. Immature inflorescences were preventively enclosed in organdy bags. Once anthesis began, as indicated by the opening of the spathe, each inflorescence was subjected to one of the following treatments: (1) manual pollination + wasps, (2) manual pollination only (positive control), (3) wasps only (no pollination), and (4) no pollination and no wasps (negative control). Manual pollination was necessary because the organdy bags prevented natural pollination of the flowers. The ‘pollination + wasps’ treatment examined the interaction between pollination and galling. The ‘pollination only’ treatment evaluated the role of pollination in preventing inflorescence abscission. The ‘wasps only’ treatment investigated the galling process in the absence of pollination, while the ‘no pollination and no wasps’ treatment assessed the inherent rate of inflorescence abscission. Treatments were randomly assigned to each inflorescence by first attributing a unique number to each one and then using the ‘sample’ function in the R programming environment [44] to ensure randomization.

For the controlled pollination, pollen was collected 1–2 days in advance from different *T. bipinnatifidum* individuals and stored in 10 mL glass flasks at 4 °C until use. In the experimental inflorescences, a generous amount of pollen was applied to the pistillate flower’s stigmata using a fine brush.

For the oviposition treatments, ripe infructescences containing galls with wasps nearing emergence were collected in advance from different *T. bipinnatifidum* individuals. The infructescences were enclosed in organdy bags under laboratory conditions until the adult wasps emerged. To keep the wasps alive, they were fed with a 10% sucrose solution on dampened cotton balls ad libitum, and mating was allowed within the bags. Only female wasps were selected for the experiment using an entomological aspirator. Approximately 30 female wasps were introduced into each bagged inflorescence for the oviposition treatments, where they were allowed to oviposit in the pistillate flowers. In treatments that combined pollination and wasp oviposition, manual pollination was performed first, followed by the introduction of the wasps. This experiment was repeated on nine different *T. bipinnatifidum* individuals, totaling 91 manipulated inflorescences.

The inflorescences were monitored every 4–5 days, from the day the treatments were applied until the dehiscence line at the base of the spathe became evident in the retained inflorescences. Inflorescence abscissions during the monitoring period were recorded. The ripe infructescences were collected and individually enclosed in organdy bags for wasp emergence. For all inflorescences and infructescences, we quantified the total number of flowers or fruits, the total number of flowers or fruits containing galls, the total number of fruits containing seeds, and the total number of fruits containing both seeds and galls.

The treatment results for each variable were compared by ANOVA using the R version 4.4.2 programming environment. We plotted the model residuals against the quantiles of the standardized normal distribution and the expected values. No substantial deviations from the ANOVA assumptions of residual normality and variance homogeneity were detected.

### 4.3. Oviposition Observations

To document the oviposition behavior of *A. gallicola*, we conducted field observations of female wasps interacting with receptive inflorescences of *T. bipinnatifidum*. Observations included both free-living wasps in natural settings and introduced wasps under experimental conditions. Photodocumentation was performed using a Canon S5 camera with a Raynox macro lens (Tokyo, Japan). To identify the pistillate flowers where eggs were deposited, we immediately killed any female observed during the oviposition process by applying a droplet of chloroform. In the laboratory, individual flowers containing the ovipositing wasp were carefully detached and dissected under a stereoscope at 40× magnification to locate the wasp egg within each flower. We dissected approximately 15 flower ovaries. The dissected flowers were photographed using a Leica MZ16 stereomicroscope coupled with a digital camera (Wetzlar, Germany).

### 4.4. Larva and Gall Development

The gall and larval development study was conducted on two inflorescences exposed to wasp oviposition without pollination. Immature inflorescences were preventively enclosed in organdy bags. At the beginning of the anthesis, 30 female wasps obtained from a different *T. bipinnatifidum* plant were introduced into each bag. Five developing fruits were sampled every five days using fine forceps, starting from the day the inflorescence closed until the larvae completed their development. To sample these galls without removing the entire inflorescence, a door-like 10 cm × 5 cm incision was made at the base of the spathe to gain access to the inflorescence. This incision was used for all subsequent sampling during gall development. After each sampling, the door-like cut was repositioned in the spathe and secured with a metal wire around it. The inflorescence remained enclosed in the organdy bag throughout the study to prevent insects and other small animals from accessing it.

The sampled fruits were fixed in FAA 50 (formalin: acetic acid: alcohol 50% [45]) for 24 h and then stored in 70% ethanol. Of the five fruits collected per sampling, two to three were dissected under a stereomicroscope to locate the immature stages of the wasps. Images of the dissected material and larvae were captured using a Leica MZ16 stereomicroscope coupled with a digital camera. Body length measurements of all extracted immature stages were taken using the Leica^TM^ Application Suite version 3.3.0 software. The remaining sampled fruits were reserved for histological study. The material was processed following standard dehydration and softening protocols, embedded in Leica Historesin^®^ [46], and sectioned into 5–6 µm slices using a Leica RM 2245 microtome. Serial sections were stained with 0.05% toluidine blue, pH 4.4 [47], and mounted on slides. Histological slides were photographed using a digital camera attached to a Leica DM 4500 microscope. All histological slides and wasp samples are held by R.A.S. Pereira as voucher material.

## Figures and Tables

**Table 1:** Percentage of inflorescence abscission in *Thaumatophyllum bipinnatifidum* under different wasp oviposition/pollination treatments. The total number of inflorescences per treatment is shown in parentheses. Treatments sharing the same letter did not differ based on chi-squared pairwise comparisons with Bonferroni correction at the 5% significance level.

Treatment (Wasp/Pollination)	Percentage of Abscission
+/+	33.0 (15) ^a^
+/−	64.3 (28) ^a^
−/+	33.0 (24) ^a^
−/−	100 (24) ^b^

**Table 2:** Mean number (±standard deviation) of galls and seeds per inflorescence or infructescence in *Thaumatophyllum bipinnatifidum* across different treatment combinations. *n* is the number of examined inflorescences, and NA indicates the treatments for which, by definition, the data were not available.

Variable	Treatments (Wasp/Pollination)			ANOVA
	+/+	+/−	−/+	
Fruits (total)	313.5 ± 110.4	231.2 ± 60.7	265.3 ± 115.4	*F*_2,33_ = 1.652, *p* = 0.207
Fruits with only seeds	125.5 ± 112.7	NA	235.4 ± 99.3	*F*_1,24_ = 6.8, *p* = 0.015
Flowers with only galls	123.0 ± 109.7	206.3 ± 57.9	NA	*F*_1,18_ = 4.51, *p* = 0.048
Fruits with galls + seeds	32.3 ± 37.9	NA	NA	-
Empty flowers	32.7 ± 33.3	24.9 ± 19.4	29.9 ± 29.2	*F*_2,33_ = 0.198, *p* = 0.821
*n* *	10	10	16	-

* Due to inflorescence abscission, the treatment results were unbalanced across the experiments conducted on the nine *T. bipinnatifidum* individuals. Consequently, a mixed-effects model approach could not be applied to the data analysis. Therefore, *n* represents a combination of biological and technical replicates.

## Data Availability

The data supporting the findings of this study are available from the corresponding authors upon reasonable request. The data are not publicly available because they form part of a larger ongoing research project, and public dissemination at this stage could compromise the ability to develop and publish further findings.
